# Design of a Humidity Sensor Tag for Passive Wireless Applications

**DOI:** 10.3390/s151025564

**Published:** 2015-10-07

**Authors:** Xiang Wu, Fangming Deng, Yong Hao, Zhihui Fu, Lihua Zhang

**Affiliations:** 1 School of Electrical and Electronic Engineering, East China JiaoTong University, Nanchang 330013, China; E-Mails: zgxiangyu@ecjtu.edu.cn (X.W.); daiwei@ecjtu.edu.cn (Z.F.); lhzhang@ecjtu.edu.cn (L.Z.); 2 School of Mechatronic Engineering, East China JiaoTong University, Nanchang 330013, China; E-Mail: haoyong@ecjtu.edu.cn

**Keywords:** humidity sensor, complementary metal oxide semiconductor (CMOS), radio frequency identification (RFID), rectifier, sensor interface

## Abstract

This paper presents a wireless humidity sensor tag for low-cost and low-power applications. The proposed humidity sensor tag, based on radio frequency identification (RFID) technology, was fabricated in a standard 0.18 μm complementary metal oxide semiconductor (CMOS) process. The top metal layer was deposited to form the interdigitated electrodes, which were then filled with polyimide as the humidity sensing layer. A two-stage rectifier adopts a dynamic bias-voltage generator to boost the effective gate-source voltage of the switches in differential-drive architecture, resulting in a flat power conversion efficiency curve. The capacitive sensor interface, based on phase-locked loop (PLL) theory, employs a simple architecture and can work with 0.5 V supply voltage. The measurement results show that humidity sensor tag achieves excellent linearity, hysteresis and stability performance. The total power-dissipation of the sensor tag is 2.5 μW, resulting in a maximum operating distance of 23 m under 4 W of radiation power of the RFID reader.

## 1. Introduction

Humidity measurement is essential for a wide range of applications in many fields including meteorology, agriculture, industrial control, medical instruments, *etc.* Humidity sensors usually measure relative humidity (RH) rather than absolute humidity. Relative humidity is the ratio of the moisture level to the saturated moisture level at the same temperature and pressure and expressed as a percentage. The main mechanisms to sense and measure RH include detecting changes in the optical [1,2], mechanical [3,4] and electrical [5,6] properties of sensing materials. The majority of the electrical humidity sensors in commercial use are of the capacitive type, which can offer lower power consumption and a less complex interface circuit compared with other types. Due to its good moisture absorption and compatibility with integrated circuit (IC) fabrication technologies, polyimide is a good candidate for moisture sensing films in capacitive humidity sensors for both micro-electro-mechanical system (MEMS) and complementary metal oxide semiconductor (CMOS) technologies [7]. Fabrication of humidity sensors in CMOS technology allows them to be easily integrated with other signal- processing circuits on a single chip, resulting in advantages including improved accuracy, reduced size and lower fabrication cost.

Radio frequency identification (RFID), as a wireless automatic identification technology, is widely applied in traffic management, logistics transportation, medicine management, food production, *etc.* [8]. Passive RFID tags offer several advantages such as battery-less operation, wireless communication capability, high flexibility, low cost and fast deployment, which all result in their extensive use in commercial applications [9]. The passive RFID tag collects the radiation energy from the RFID reader as its power supply. Hence, the power dissipation of the passive RFID tag, which determines the maximum operating distance of the tag, is crucial for the design of a passive RFID tag. Recently, with the rapid development of the Internet of Things and sensor technology, research on adding sensing functionality to RFID tag is become a hot topic [10,11,12]. This smart RFID sensing tag not only extends the application field of RFID, but also contributes to reduce the fabrication cost of RFID systems.

Previous research on capacitive CMOS humidity sensors was mainly focused on the humidity sensor element [13,14,15,16,17]. However the designs in [13,14,15,16] require some post-processing steps, the design in [17] uses materials and steps not commonly found in standard fabrication processes, which all resulting in increased fabrication costs. Benefiting from advantages such as lower packaging cost, smaller parasitic capacitance, smaller chip area, *etc*., fully integrated humidity sensors were recently introduced [18,19,20]. The designs in [18,19] reported humidity sensors with integrated interfaces. The design in [20] reported a wireless humidity sensor, but its operating frequency is 13.56 MHz which is only suitable for low speed and short distance applications.

Our motivation in this work has been to develop a wireless humidity sensor operating at 900 MHz frequency. For low-cost applications, the humidity sensor element is incorporated with the wireless transceiver blocks and the humidity sensor element is fabricated in standard CMOS technology without any post-processing. An ultra-low power sensor interface is introduced to ensure that the wireless sensor can work in passive mode. The rest of the paper is organized as follows: Section 2 presents the system architecture of the wireless humidity sensor and then the analysis of the design of key blocks is detailed in Section 3. Section 4 illustrates the measurement results and then conclusions are presented in Section 5.

## 2. System Design

The operating frequency plays a very important role in RFID systems. In general, the operating frequency defines the data rate of the system and the tag size. Ultra-high frequency (UHF) RFID systems are a better solution for next generation auto-ID applications because they have advantages including fast transmission speed, long communication distance and simple modulation schemes. The theoretical practicable operating power of an RFID tag *P_t_* is calculated from the Friis transmission equation [21]:

(1) $$P_{t}=E_{r}\cdot G_{a}\cdot \eta_{r}\cdot {\left(\frac{\lambda }{4\pi d}\right)}^{2}$$

where *E_r_* is the effective isotropic radiation power of a reader, *G_a_* is the tag antenna gain, *η_r_* is the RF-to-DC power conversion efficiency of the rectifier, and *d* is the communication distance. From this equation, it can be concluded that in order to achieve a longer communication distance, lower tag power and higher *η_r_* of the rectifier are critical for the UHF RFID tag design because *E_r_* is limited by regional regulations (4 W is the maximum transmitted power) and *G_a_* is roughly determined by the allowable antenna area (1.64 for the λ/2 dipole antenna).

**Figure 1:**
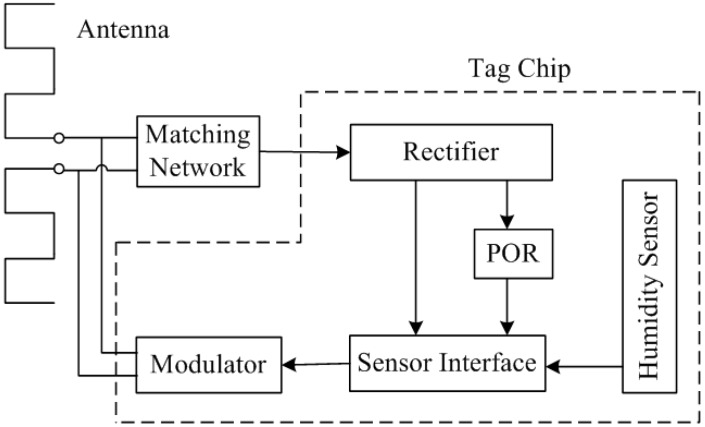
Architecture of the proposed wireless sensor.

Figure 1 shows the architecture of the proposed passive wireless humidity sensor. The blocks shown, except for the antenna and matching network, are integrated on a single tag chip. The tag antenna, which is matched with the tag chip through the matching network, receives the electromagnetic waves from the RFID reader. The rectifier multiplies and transfers the received RF signal to a DC supply voltage for the subsequent circuitry. Once the output of the rectifier reaches the operating voltage, the power-on-reset (POR) block generates a reset signal for the sensor interface. Due to the good immunity to voltage supply, the regulator block in the conventional RFID tag architecture is not employed in this proposed architecture, which can be concluded in Section 3.3. The demodulator block is also not included in this architecture, which means that this wireless sensor will operate without any addressing as long as the sensor tag receives enough large signals from the RFID reader [22].

**Figure 2:**
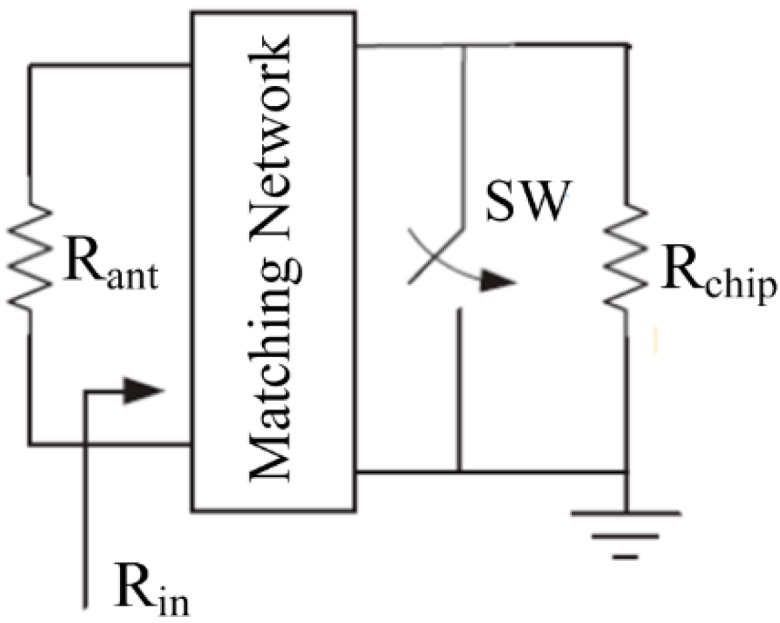
ASK backscattering scheme.

For low-cost and small-size applications, the meandered dipole antenna is a natural choice for UHF RFID taga [23]. The modulator of the tag chip employs a low-power backscattering scheme, in which the RFID tag acts as a reflector that reflects the incident RF wave back to the RFID reader. Backscattering can be either amplitude shift keying (ASK) or phase shift keying (PSK) backscattering. ASK backscattering is much more simple and efficient than PSK backscattering [24], therefore this work employs an ASK backscattering scheme. Figure 2 shows the scheme of the ASK-backscattering modulation. If the tag intends to send digital “1” to RFID reader, the SW is opened which leads to the perfect match (*R_in_* = *R_ant_*) and then the tag entirely absorbs the electromagnetic wave from RFID reader. If the tag intends to send a digital “0” to the RFID reader, the SW is closed which leads to the complete mismatch (*R_in_* = *0*) and then the tag entirely backscatters the electromagnetic signal to the reader.

## 3. Key Blocks Design

### 3.1. Humidity Sensor

The reported CMOS humidity sensor designs [13,14,15,16] require some post-processing steps and the design [17] uses materials and steps not commonly found in standard fabrication processes, all of which will undoubtedly increase the fabrication cost. For a CMOS process, interdigitated top metal fingers, with polyimide filled into the finger gaps, can be utilized for capacitive humidity sensing [18,19,20]. The line-to-line coupling capacitance of the top metal is sensitive to the dielectric constant of the filling material. Furthermore, due to the high precision of photolithography, such a kind of sensing structure is highly reproducible with less inter-die variations.

**Figure 3:**
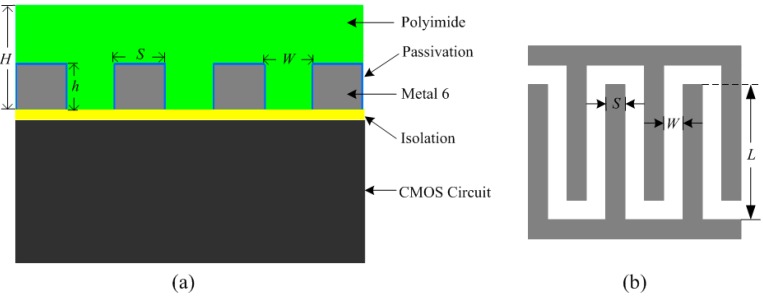
Proposed humidity sensor: (**a**) humidity sensor structure and (**b**) top view of the humidity sensor.

Figure 3 illustrates the structure of the proposed capacitive humidity sensor in a standard CMOS process, resulting in the integration with other tag blocks on a single chip. The proposed humidity sensor was fabricated in the TSMC 0.18 μm 1P6M CMOS process. The top metal layer (Metal 6) was deposited and patterned with standard optical lithography and wet etching over the isolation layer to form the interdigitated structure. The sensing capacitor was then covered with a humidity-sensitive polyimide layer. As seen from Figure 3, *L* is the length of metal electrodes, *S* is the width of each electrode, and *W* is the distance between adjacent electrodes. The thickness of the polyimide layer is *H*, which is generally larger than metal thickness *h*. For an *N* finger array sensor, the total sensor capacitance can be expressed as [25]:

(2) $$C_{sensor}=N{\text{ε}}_{wet}\frac{Lh}{W}$$

The metal thickness *h* and the electrode width *S* are limited by the CMOS process. The thickness of the polyimide layer *H* is a trade-off of between the sensitivity and response time. The selection of *H* has great influence on the sensitivity and response time of the sensor. Generally, a humidity sensor with a thicker polyimide layer has better sensitivity and longer response times. Considering the factors including sensor capacitance, chip area sensitivity and *etc.*, this work chooses *N* = 40, *L* = 200 µm, *W* = 2.5 µm, *S* = 2.5 µm, *h* = 1 µm and *H* = 2 µm.

### 3.2. Rectifier

A popular performance metric of a rectifier is its power conversion efficiency which is defined as:

(3) $$\eta_{r}=\frac{P_{out}}{P_{in}}$$

where *P_out_* is the average DC output power generated at the output of the rectifier and *P_in_* is the average RF power available at the input of the rectifier. From a circuit-level point of view, *η_r_* is mainly degraded due to the forward drop of the switch diodes or transistors [26]. There are several techniques that are used to reduce the turn on voltage, including Schottky diodes, low *V_th_* transistors and dual-poly floating gate transistors, which all require advanced CMOS processing at an additional cost [27,28]. In standard CMOS technology, a diode-connected MOS transistor is usually employed as switch transistor, but unfortunately the high threshold-voltage of MOS switch will greatly deteriorate the *η_r_* of this architecture. The classic differential-drive architecture [29] is an excellent solution for high-efficiency and low-cost applications, however the *η_r_* curve of this design drops rapidly on the two sides of the optimal point.

**Figure 4:**
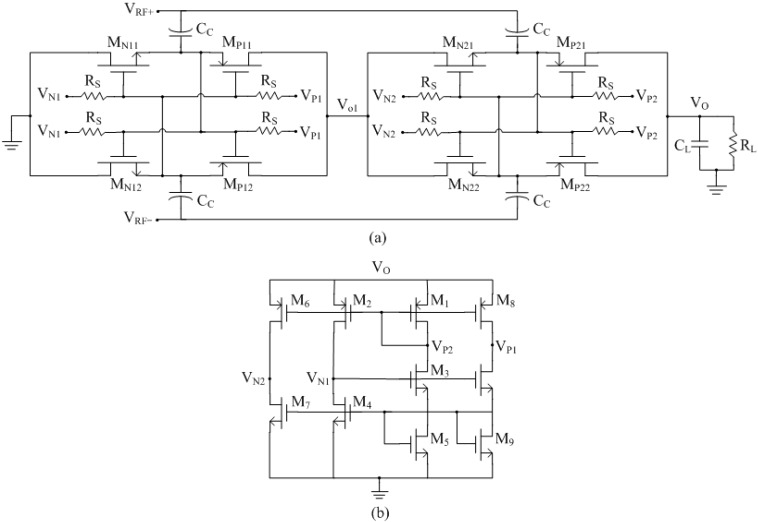
Schematic of the proposed rectifier: (**a**) two-stage rectifier and (**b**) bias-voltage generator.

Inspired by Kamalinejad [30], this work employed a gate-boosting scheme to achieve a flat *η_r_* curve. When the input power is smaller (or larger) than the optimal point of the *η_r_* curve, an extra bias-voltage is added to positively (or negatively) boost the effective gate-source voltage of the switches. When input power is at the optimal point, the extra bias-voltage equals to zero. Thus, the bias-voltage is dynamic. According to the rules discussed above, Figure 4a shows the schematic of the proposed rectifier, which consists of two identical stage. The NMOS transistors M_N11-22_ and PMOS transistors M_P11-22_ form the differential-drive switch. The bias-voltage V_N1,2_ and V_P1,2_ are employed to boost the gate-source voltage of NMOS switch and PMOS switch, respectively. The large resistor *R_S_* is added to block the AC component of V_N1,2_ and V_P1,2_. In CMOS process, *R_S_* could be replaced by a PMOS transistor that operates in the cut-off region to avoid the large silicon area. Figure 4b shows the schematic of the dynamic bias generator circuit, which is directly supplied by the output voltage of the rectifier V_O_. The transistors M_1_-M_5_ form the core of the reference voltage generator and the other transistors form the current mirrors. The required bias-voltage V_N1,2_ and V_P1,2_ can be achieved through by proper sizing of transistors.

### 3.3. Sensor Interface Design

The capacitive humidity sensor acts as a capacitor when it works with the sensor interface. There are several popular techniques to perform the capacitance-to-digital conversion function. The traditional conversion starts with a capacitance-to-voltage converter, which is then followed by a voltage-to-digital converter [31,32]. This technique can achieve high speed and high resolution performance, however, due to the use of an operational amplifier in switched-capacitor amplifiers (SCA), this technique results in too much power dissipation in the mW range. Accordingly, the inverter is proposed to replace the operational amplifier in SCA [18,33], resulting in a significant reduction of the entire power dissipation. Nevertheless, they still employ a relatively high supply voltage. Another conversion method in the time domain adopts pulse-width modulation for low-power applications [34,35]. This technique is suitably applied in the field of large-scale capacitance variation.

**Figure 5:**
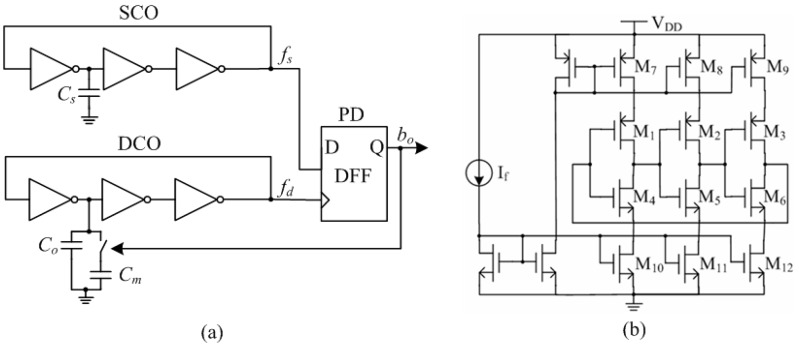
Proposed capacitive sensor interface: (**a**) architecture and (**b**) schematic of current-starved oscillator.

This work employs a phase-locked loop (PLL)-based architecture which can directly achieve the capacitance-to-digital conversion [36]. The proposed sensor interface is shown in Figure 5a. It consists of three blocks, including sensor-controlled oscillator (SCO), digital-controlled oscillator (DCO) and phase detector (PD). Both the SCO and the DCO are implemented as three-stage inverter-based ring oscillators. The sensor capacitor *C_s_* acts as the variable load on a single stage of the SCO, thereby generating a sensor-controlled frequency *f_s_*. The DCO is steered by the PD output signal *b_o_*, which is a representation of the phase difference between the SCO and the DCO. The variable capacitive load on a single stage of the DCO consists of two capacitors, *C_o_* and *C_m_*. The capacitor *C_o_*, designed equal to the quiescent value of *C_s_*, always connected to the DCO. But the capacitor *C_m_*, designed slightly larger than the maximum variation of *C_s_*, is swapped in or out of the DCO depending on the feedback from the PD. The PD is simply composed of a single-bit d-flip-flop. When the entire feedback loop is locked, the average digital frequency *f_d_* will correspond to the sensor frequency *f_s_*. Therefore, *b_o_* represents the digital value of the sensor capacitance. This paper employs current-starved ring oscillators for the design of SCO and DCO. As shown in Figure 5b, M_1_-M_6_ form the 3-stage inverter-based ring oscillator, which current is constrained by the current mirror M_7_-M_12_. Although the extra transistors of M_7_-M_12_ need a higher supply voltage, the current flowing through the inverters can be constrained much smaller, resulting in lower power-dissipation and higher temperature stability.

For this work, the humidity sensor’s capacitance varies from 5 to 6.5 pF within the relative humidity range. Hence the capacitor *C_o_* and *C_m_* are selected as 5 pF and 2 pF respectively. Due to the fully digital architecture, the power supply of the interface is selected as 0.5 V. This architecture has an inherent immunity to supply voltage variations due to the fact that the digital output *b_o_* is determined by the phase difference between the DCO and the SCO, resulting in the exclusion of a voltage regulator in the proposed wireless humidity sensor tag (seen in Figure 1).

## 4. Measurements Result and Discussion

**Figure 6:**
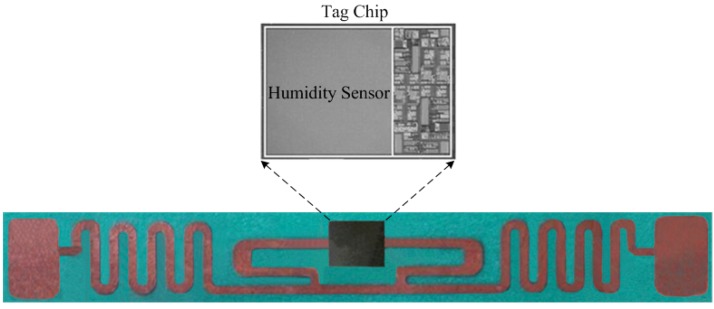
Photo of the proposed wireless humidity sensor.

**Figure 7:**
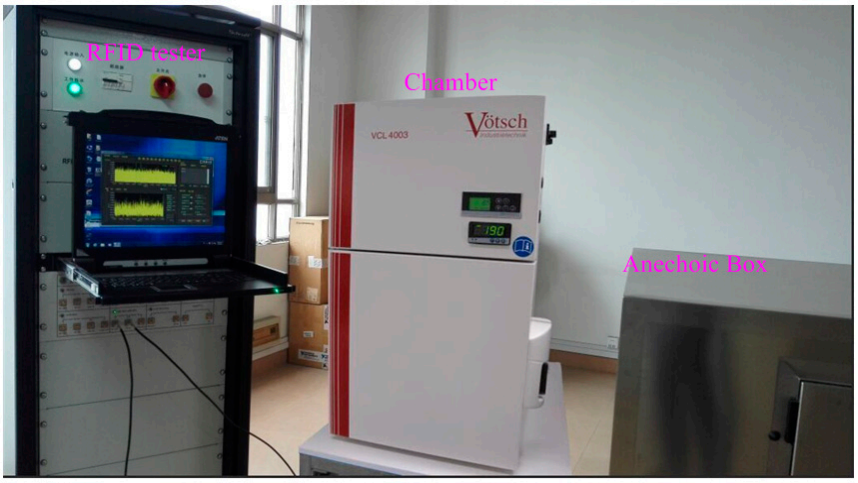
Measurement environment.

Figure 6 shows the proposed wireless humidity sensor, which was fabricated in the TSMC 0.18 μm CMOS process. The sensor tag equipped with a UHF antenna occupies an area of 80 × 6 mm^2^ and the packaged sensor tag chip covers around 7 × 3 mm^2^. The humidity sensor performance was measured in a Votsch VCL4003 temperature and humidity chamber. As shown in Figure 7, the wireless test environment consists of a special RFID test instrument, VISN-R1200 from VI Service Network and an anechoic box. The VISN-R1200 can work as an adjustable RFID reader and display the testing signals simultaneously. The RFID tag was tested in the anechoic box for the electromagnetic shielding.

**Figure 8:**
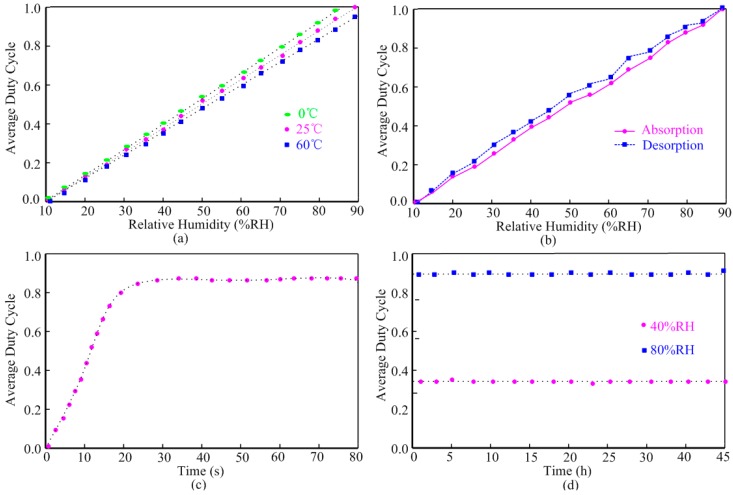
Measured humidity sensor performance: (**a**) linearity; (**b**) hysteresis; (**c**) response time and (**d**) stability.

Figure 8 shows the measured humidity sensor performance. Figure 8a illustrates the average duty cycle responses of the interface output with respect to the relative humidity at different temperature.

Within the relative humidity range from 10% to 90% RH, this humidity sensor achieves highly linear performance. Due to the temperature dependence of the dielectric constant of polyimide, the average duty cycle shows an acceptable maximum temperature variation of 10% from 0 to 60 °C. The humidity sensor achieves a sensitivity of 18.75 fF/%RH at 25 °C. The hysteresis performance of the sensor at 25 °C is shown in Figure 8b. The maximum difference between the moisture absorption and desorption at the point 55% RH is not exceeding 8%. Figure 8c shows that the response time of the humidity sensor is 20 s, which was measured to 90% point of the final steady state after an abrupt relative humidity change from 10% RH to 80% RH at 25 °C. The proposed humidity sensor was then tested for 45 h at the humidity of 40% RH and 80% RH respectively. As shown in Figure 8d, the measurement results showed excellent stability and no obvious drift was observed. Table 1 compares the proposed humidity sensor with previous designs. Our work achieves an average sensitivity, but it is fabricated without post-processing steps and integrates wireless transceiver blocks.

**Table 1:** Comparison of integrated humidity sensors.

Design	Sensor Structure	Process	Sensitivity	Fabrication Post-Processing	On-Chip Readout Circuit
[13]	Interdigitated	3 µm	5 fF/%RH	Yes	No
[15]	Parallel Plate	0.5 µm	303 fF/%RH	Yes	No
[16]	Interdigitated	0.35 µm	0.11 MHz/%RH	Yes	No
[17]	Woven Mesh	0.15 µm	1.78 mV/%RH	No	Yes
[18]	Interdigitated	0.16 µm	7.43 fF/%RH	No	Yes
[20]	Interdigitated	0.6 µm	30 fF/%RH	No	Yes
This work	Interdigitated	0.18 µm	18.75 fF/%RH	No	Yes

**Figure 9:**
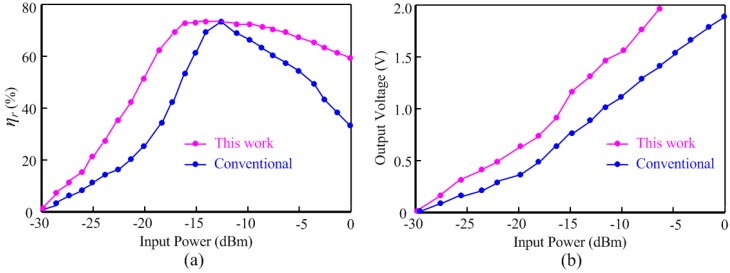
Performance comparison between conventional rectifier and this work: (**a**) power conversion efficiency and (**b**) output voltage.

For a 900 MHz input frequency and 50 kΩ load, the *η_r_* curve of the proposed rectifier is shown in Figure 9a. As compared to the conventional differential-drive rectifier [29], the proposed rectifier shows a flat *η_r_* curve. When input power is 13 dBm, the two curves both reach the optimal point 69%.

As for the input power range which *η_r_* is above 60%, the proposed rectifier and the conventional rectifier achieve −19 dBm and −7 dBm range, respectively. Figure 9b compares the output voltage of the proposed rectifier with that of the conventional rectifier for a 50 kΩ load at 900 MHz input frequency. The proposed scheme provides an output voltage of 0.6 V at the low input power of −20 dBm, while this output for the conventional counterpart is 0.3 V which is insufficient for many RFID applications.

The sensor interface performance was measured under a constant 0.5 V supply voltage from the rectifier. The performance comparison with previous designs is shown in Table 2. Owing to its simple architecture, the proposed sensor interface covers a reduced chip area and can operate on a 0.5 V ultra-low supply voltage. Despite the moderate effective number of bits (ENOB), this interface reduces power consumption significantly in contrast with the previous designs, making it especially suitable for passive RFID applications.

**Table 2:** Performance comparison of capacitive sensor interfaces.

Interface	Process (µm)	Supply (V)	Area (mm^2^)	ENOB (bits)	FOM (pJ/conv)	Power (µW)
[18]	0.16	1.2	0.15	12.5	8300	10.3
[33]	0.09	1.0	N/A	10.4	1.4	3.0
[34]	0.35	3.0	0.09	9.3	3.4	54.0
[35]	0.32	3.0	0.52	9.8	4.5	84.0
This work	0.18	0.5	0.01	6.8	1.6	1.1

**Figure 10:**
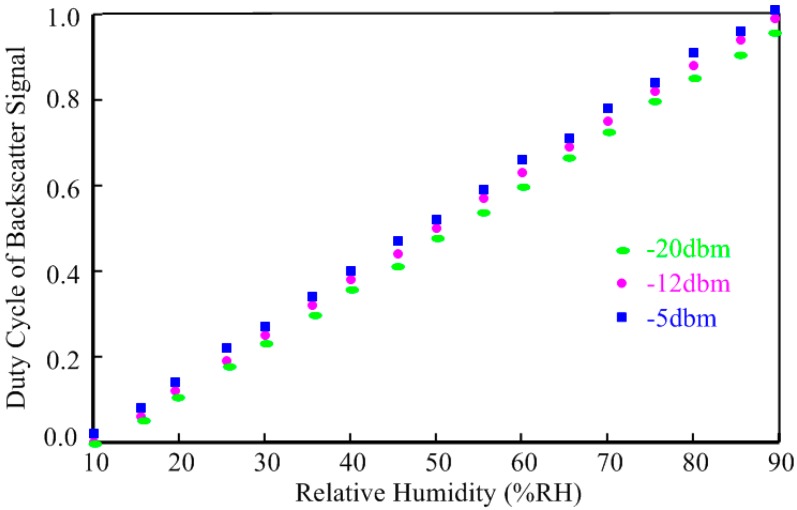
Duty cycle of backscatter signal *versus* relative humidity.

The measured minimum power dissipation of the wireless humidity sensor tag is 2.5 µW, resulting in a maximum operating distance of 23 m under 4 W RFID reader radiation power conditions. Finally, the measured backscattering performance of the sensor tag is shown in Figure 10. The measurement as repeated when the input power is −20 dbm, −12 dbm and −5 dbm, respectively. Due to the interface’s immunity to the supply voltage, the three measured results show excellent linearity and consistency.

## 5. Conclusions

We have developed a humidity sensor tag for wireless applications. This wireless humidity sensor tag operates in the UHF band and employs an ASK modulator. The humidity sensor element is designed in standard CMOS technology without any post-processing, which results in integration with other tag blocks and low fabrication cost. A two-stage rectifier adopts a dynamic bias-voltage generator to boost the effective gate-source voltage of the switches in differential-drive architecture, resulting in a flat power conversion efficiency curve. The sensor interface, based on phase-locked loop theory, employs a fully-digital architecture. Despite the moderate ENOB, this interface can operate on ultra-low supply voltage and then reduces the power dissipation significantly compared to previous designs. The total power-dissipation of the sensor tag is 2.5 µW, resulting in a maximum operating distance of 23 m under 4 W RFID reader radiation power conditions.
